# 838. The Effect of Obesity on Mortality and Hospital Length of Stay Among Severe Burns Patients

**DOI:** 10.1093/jbcr/irag033.277

**Published:** 2026-04-06

**Authors:** Amir Khorrami, Aman Sharma, Sara Sheikh-Oleslami, Ou Jia (Emilie) Wang, Anthony Papp

**Affiliations:** Vancouver General Hospital BC Burn Unit, Vancouver, BC; Vancouver General Hospital BC Burn Unit, Vancouver, BC; The University of British Columbia, Vancouver, BC; Vancouver General Hospital BC Burn Unit, Vancouver, BC; Vancouver General Hospital BC Burn Unit, Vancouver, BC

## Abstract

**Introduction:**

Obesity, defined as a body mass index (BMI) greater than 30 kg/m2, has been associated with increased surgical complications. However, numerous studies in trauma and critical illness literature have demonstrated an “obesity paradox,” whereby elevated BMI appears to have a protective effect on mortality. It has been hypothesized that increased BMI reduces mortality due to the increased metabolic and nutritional reserves, as well as the immunologic benefits of adipose tissue.

Severe burns, defined as total body surface area (TBSA) ≥20%, present a multifaceted challenge to healthcare providers, with mortality influenced by factors including age, gender, TBSA, burn depth, and presence of inhalation injury. Burn literature shows conflicting evidence on the association between obesity and mortality. This study aims to evaluate the relation between obesity and mortality in patients with severe burns at a single burn centre.

**Methods:**

This is a single-centre, consecutive, non-randomized retrospective chart review of all adult patients (age > 18) with total body surface area (TBSA) > 20% who were admitted at our burn centre between October 2019 to July 2024.

Inclusion criteria were (i) patients older than 18 years, (ii) Diagnosis of major burn injury requiring admission (TBSA>20%), and (iii) collected BMI data.

Non parametric Wilcoxon rank test and Fisher’s exact tests were used to compare demographic data, burn characteristics, and mortality. The primary outcome (mortality) was binary and was analyzed using a logistic regression model.

**Results:**

There were 82 patients (59 male, 23 female) who met the inclusion criteria and were included in the final analysis. Patients were divided into 3 BMI groups (normal weight, overweight, obese). There were no statistical differences between the BMI groups with regards to age (*p*=.21), TBSA (*p*=.09), and presence of inhalational injury (*p*=.89). Overall mortality was 15% (12/82). The most common comorbidity was polysubstance use disorder, noted in 61% (50/82) of patients.

There was a significant statistical difference in mortality between the overweight group (24.2%) compared with the normal weight group (6.3%) (*p*<.001) and obese group (12.5%) (*p*=.05). However, there was no statistically significant difference in mortality between normal weight and obese groups (*p*=.20). A multivariate logistic regression model found that numeric BMI was not significant for impacting mortality (*p*=.41). With regards to patients that survived, there was a trend towards shorter hospital stay with increased mortality, however this did not meet statistical significance.

**Conclusions:**

We did not observe the classic obesity paradox with regards to burn mortality in our centre. However, in patients who survived their burn injury, increased BMI was clinically significant for shorter hospital stay.

**Applicability of Research to Practice:**

Further research is required to incorporate BMI as an independent predictive outcome for burn patients.

**Funding for the study:**

N/A.

